# Mutational analysis of driver genes defines the colorectal adenoma: in situ carcinoma transition

**DOI:** 10.1038/s41598-022-06498-9

**Published:** 2022-02-16

**Authors:** Jiri Jungwirth, Marketa Urbanova, Arnoud Boot, Petr Hosek, Petra Bendova, Anna Siskova, Jiri Svec, Milan Kment, Daniela Tumova, Sandra Summerova, Zdenek Benes, Tomas Buchler, Pavel Kohout, Tomas Hucl, Radoslav Matej, Ludmila Vodickova, Tom van Wezel, Pavel Vodicka, Veronika Vymetalkova

**Affiliations:** 1 Institute of Biology and Medical Genetics, Institute of Physiology, 1st Faculty of Medicine, Charles University, Albertov 4, 128 00 Prague, Czech Republic; 2 Department of Surgery, Weiden Clinic, Söllnerstraße 16, 92637 Weiden in der Oberpfalz, Germany; 3 Department of Molecular Biology of Cancer, Institute of Experimental Medicine of the Czech Academy of Sciences, Videnska 1083, 142 00 Prague, Czech Republic; 4 Department of Pathology, Leiden University Medical Center, Leiden, The Netherlands; 5 Biomedical Center, Faculty of Medicine in Pilsen, Charles University, Alej Svobody 76, 323 00 Pilsen, Czech Republic; 6 Institute of Molecular Genetics of the Czech Academy of Sciences, Videnska 1083, 142 20 Prague, Czech Republic; 7 Department of Radiotherapy and Oncology, Third Faculty of Medicine, Charles University, Srobarova 50, 100 34 Prague 10, Czech Republic; 8 Second Department of Internal Medicine, Third Faculty of Medicine, Charles University, Srobarova 50, 100 34 Prague 10, Czech Republic; 9 DT Gastroenterology, Roskotova 1/1225, Prague 4, Czech Republic; 10 Department of Internal Medicine, Third Faculty of Medicine Charles University and Thomayer University Hospital, Ruska 87, 100 00, Prague, Czech Republic; 11 Department of Oncology, First Faculty of Medicine, Charles University and Thomayer University Hospital, Videnska 800, 140 59 Prague, Czech Republic; 12 Department of Hepatogastroenterology, Institute for Clinical and Experimental Medicine, Videnska 1958/9, 140 21 Prague, Czech Republic; 13 Department of Pathology and Molecular Medicine, Third Faculty of Medicine, Charles University and Thomayer University Hospital, Videnska 800, 140 59 Prague, Czech Republic; 14 Department of Pathology, Third Faculty of Medicine, Charles University and University Hospital Kralovske Vinohrady, Srobarova 50, 100 34 Prague 10, Czech Republic

**Keywords:** Cancer genetics, Cancer epigenetics

## Abstract

A large proportion of colorectal carcinomas (CRC) evolve from colorectal adenomas. However, not all individuals with colonic adenomas have a risk of CRC substantially higher than those of the general population. The aim of the study was to determine the differences or similarities of mutation profile among low- and high-grade adenomas and in situ carcinoma with detailed follow up. We have investigated the mutation spectrum of well-known genes involved in CRC (such as *APC, BRAF, EGFR, NRAS, KRAS, PIK3CA, POLE, POLD1, SMAD4, PTEN,* and *TP53*) in a large, well-defined series of 96 adenomas and in situ carcinomas using a high-throughput genotyping technique. Besides, the microsatellite instability and *APC* and *MLH1* promoter methylation were studied as well. We observed a high frequency of pathogenic variants in the studied genes. The *APC*, *KRAS* and *TP53* mutation frequencies were slightly lower in adenoma samples than in in situ carcinoma samples. Further, when we stratified mutation frequency based on the grade, the frequency distribution was as follows: low-grade adenoma—high-grade adenomas—in situ carcinoma: *APC* gene 42.9–56.0–54.5%; *KRAS* gene 32.7–32.0–45.5%; *TP53* gene 8.2–20.0–18.2%. The occurrence of *KRAS* mutation was associated with the presence of villous histology and methylation of the *APC* promoter was significantly associated with the presence of *POLE* genetic variations. However, no association was noticed with the presence of any singular mutation and occurrence of subsequent adenoma or CRC. Our data supports the multistep model of gradual accumulation of mutations, especially in the driver genes, such as *APC*, *TP53* and *KRAS*.

## Introduction

Colorectal cancer (CRC), the third most common cancer and the fourth most frequent cause of cancer death worldwide^1^, represents an ideal model to investigate and dissect the genetic alterations involved in tumor initiation and progression. It has been known for some time that the majority of CRCs arises and progresses through a series of well-defined molecular and histopathological changes, the so-called adenoma-carcinoma sequence^2,3^, first described by Fearon and Vogelstein^4^.

The adenoma-carcinoma sequence was described as a gradual transformation of colorectal epithelium to adenomatous lesions and ultimately to an adenocarcinoma and a metastatic tumor. Even though most neoplastic adenomas will not give rise to cancer, it is well accepted that most colorectal carcinomas evolve from adenomatous polyps^5^. However, it cannot currently be predicted which of the early lesions will develop into cancer^6^. Molecular alterations that play a role in the initiation and progression of CRC suggest a heterogeneous adenoma-carcinoma sequence that comprises several distinct molecular pathways. These include chromosomal instability, microsatellite instability (MSI), and CpG island methylator phenotype (CIMP) pathways that all of which are responsible for genetic and epigenetic instability in CRC^2^. These genetic and epigenetic alterations affect different pathways that regulate multiple biological processes critical to cancer development^7^.

Genetic mutations enriched in both adenomas and carcinomas are likely to represent early driver events. Mutations present predominantly in the carcinomas may constitute later driver mutations involved in tumor progression. Genes mutated in adenomas and not mutated in associated carcinoma tissue comprise either random mutation events not important for cancer initiation, or rare events that were not identified in the carcinomas^8^.

The aim of the present study was to explore the genetic heterogeneity of adenomas and early carcinomas by analyzing the mutation spectrum of well-known genes involved in colorectal carcinogenesis (*APC, BRAF, EGFR, NRAS, KRAS, PIK3CA, POLE, POLD1, SMAD4, PTEN,* and *TP53*) in a large, well-defined series of adenomas and in situ carcinomas with follow up using a next generation sequencing approach.

In this context, we have analyzed each gene for the number and type of mutations present in the adenomas and in situ carcinomas and their specific relationship to MSI status. In addition, we have analyzed the levels of methylation of CRC-related genes by methylation-sensitive high-resolution melting (MS-HRM). We investigated the tumor suppressor adenomatous polyposis coli gene (*APC*), which encodes a key protein in the WNT signaling pathway and is indicated as an early event in carcinogenesis^9^, and *MLH1* gene whose aberrant promoter methylation is responsible for the loss of mismatch repair activity.

## Results

### Patient’s characteristics

The studied set included 96 patients, out of which 74 were patients with adenomas and 22 with in situ carcinoma. The clinic-pathological characteristics are presented in Table 1.

**Table 1:** Patient´s clinical characteristics.

		All n = 96 (%)	Adenomas n = 74 (%)	In situ carcinomas n = 22 (%)	*p* value (test) for difference between adenomas and in situ carcinomas
Age (mean ± SD) years		65.6 ± 10.4	65.1 ± 10.6	67.0 ± 9.9	0.48 (*t* test)
Sex	Men	58 (60.4)	46 (62.2)	12 (54.5)	0.62 (Fisher’s)
	Women	38 (39.6)	28 (37.8)	10 (45.5)	
Lesion site	Colon	54 (56.2)	46 (62.2)	8 (36.4)	0.05 (Fisher’s)
	Rectum	42 (43.8)	28 (37.8	14 (63.6)	
Polyp type	Tubular	46 (47.9)	37 (50)	9 (40.9)	0.62 (Fisher’s)
	Tubulo-villous	38 (39.6)	29 (39.2)	9 (40.9)	
	Villous	12 (12.5)	8 (10.8)	4 (18.2)	
Grade*	Low	–	49 (66.2)	–	–
	High	–	25 (33.8)	–	

*For adenoma patients only.

### APC and MLH1 promoter methylation

The promoter methylation status of *APC* and *MLH1* genes was studied by MS-HRM.

There was no remarkable difference in the distribution of promoter methylation in the *APC* gene (mean 9%): for in situ carcinoma, the mean promoter methylation in affected tissue was 8% and in adenomas it was 8.4% (9.1% for low grade and 6.8% for high grade dysplasia).

**Figure 1:**
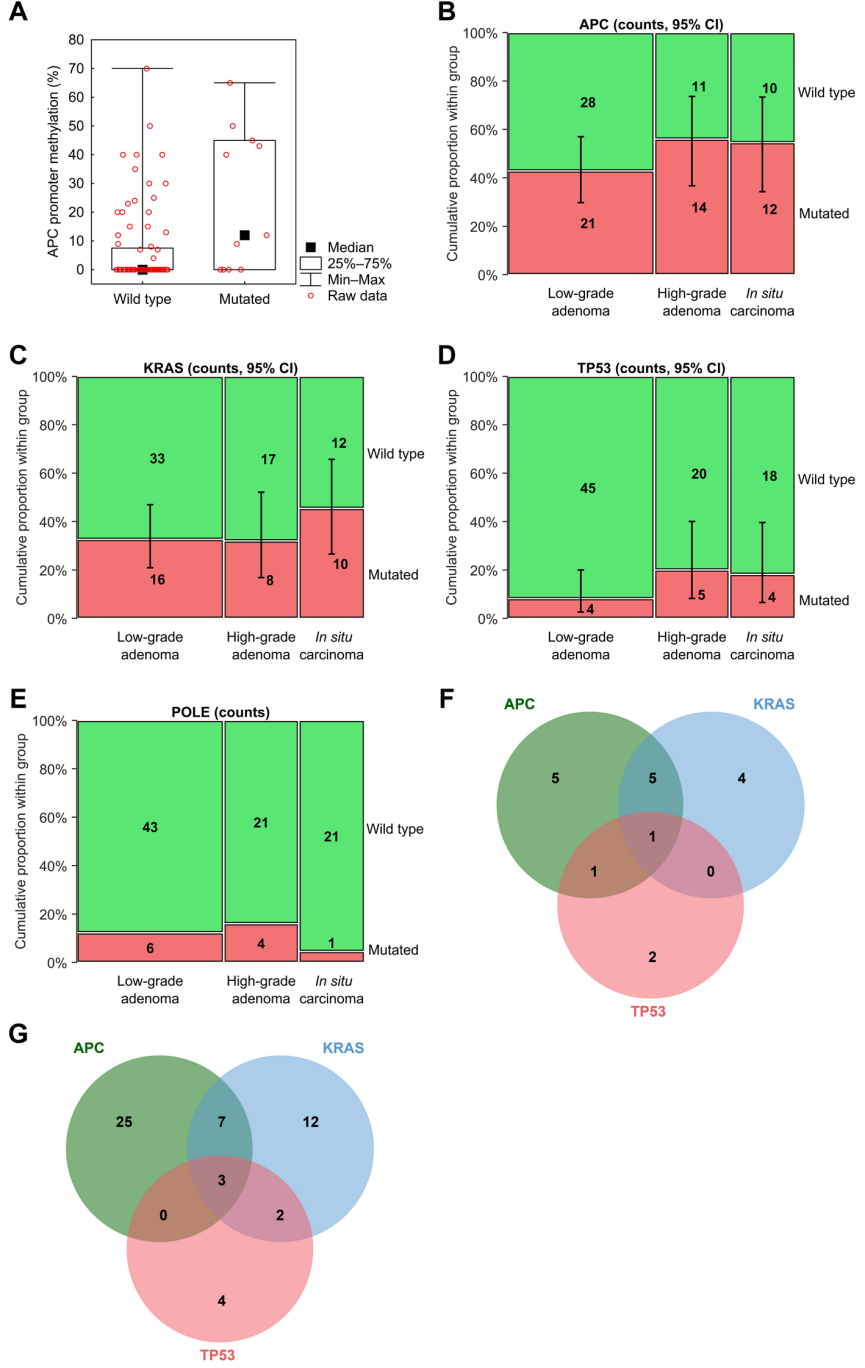
The mutated gene signature of colorectal adenomas and in situ carcinomas. (**A**) The *APC* promoter methylation distribution with *POLE* genetic variations, (**B**) The mutation distribution of *APC* gene between low-, high-grade adenomas and in situ carcinomas, (**C**) The mutation distribution of *KRAS* gene between low-, high-grade adenomas and in situ carcinomas, (**D**) The mutation distribution of *TP53* gene between low-, high-grade adenomas and in situ carcinomas, (**E**) The mutation distribution of *POLE* gene between low-, high-grade adenomas and in situ carcinomas, (**F**) The Venn diagram of mutations of *APC*, *TP53*, and *KRAS* genes in in situ carcinomas, (**G**) The Venn diagram of mutations of *APC*, *TP53*, *KRAS*, and *POLE* genes in adenomas.

Interestingly, methylation of the *APC* promoter was significantly associated with the presence of *POLE* genetic variations (*p* = 0.02, Fig. 1A; n = 11).

The only one hypermethylated *MLH1* promoter corresponded to the only sample with MSI-H status.

### MSI status

MSI status of all adenomas and in situ carcinomas was tested. In our set, only 3 samples had MSI instability: 2 of them with MSI-L and one with MSI-H status. The MSI-H status was observed in an in situ carcinoma located in the right colon while MSI-L was noticed in two low grade dysplasia samples located in the left colon.

### Mutation spectrum

In total, 96 adenomas and in situ carcinomas were analyzed for mutations in 11 genes (*APC, BRAF, EGFR, NRAS, KRAS, PIK3CA, POLE, POLD1, SMAD4, PTEN,* and *TP53*). The mutation hotspots in *KRAS, NRAS, BRAF, PIK3CA, EGFR, SMAD4* genes as well as exonuclease domain for *POLE* and *POLD1* genes were studied. Concerning the *APC*, *PTEN* and *TP53* genes, we have focused on the entire open reading frame (ORF).

**Figure 2:**
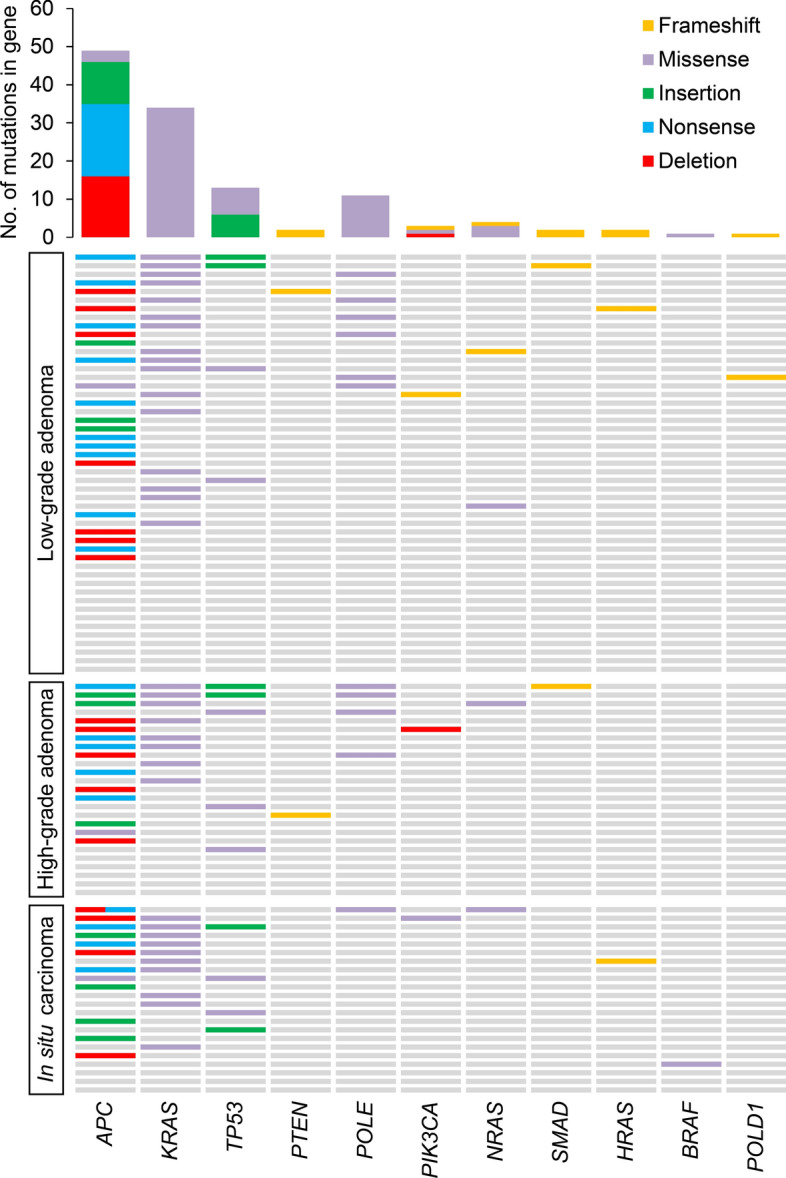
The distribution of genetic alterations detected in low-grade, high-grade adenomas, and in situ carcinomas. Each row represents a patient, and each column represents a gene. Different mutation types are indicated by different colors. The bar chart on the top shows the total number of the given gene’s mutations observed in the sample.

Of all 96 samples, 21 (18 adenomas and 3 in situ carcinomas) of them did not bear any mutation in the studied genes. Out of the remaining 75 samples with mutation(s), 56 were adenomas and 19 carcinomas (Fig. 2). We did not observe any significant differences in mutation distribution between adenomas and in situ carcinomas.

### APC

In the present study, we have focused on the entire ORF of the *APC* gene as it harbors most of the mutations described in the *APC* gene. In both the low- and high-grade adenoma samples (Fig. 1B), 36 deleterious (according to HGMD guidelines and pathogenic according to ACMG guidelines; Supplementary Table 1) mutations in 35 individuals were observed: 12 deletion (p.E1268fs, p.1275_1275del, p.D1279fs, p.T1283fs, p.1289_1291del, p.1302_1304del, p.1344_1350del, p.P1406fs, p.1431_1432del, p.T1469fs, p.P1479fs, and p.1561_1562del), 7 insertion (p.Q1226fs, p.S1316fs, p.V1359fs, p.P1391fs, p.T1469fs, p.G1481fs, and p.E1536fs), 15 nonsense (three times p.Q1273X, twice p.Q1349X, twice p.E1379X, twice p.R1432X, p.Q1276X, p.E1288X, p.S1297X, p.E1304X, p.E1335X, and p.Q1349X), and 2 missense (p.T1274M and p.E1299Q) mutations. While in carcinoma samples, 13 mutations in 12 patients were detected: 4 deletion (p.1284_1286del, p.1454_1455del, p.T1469fs, and p.E1542fs), 4 insertion (p.Q1226fs, p.M1365fs, p.S1377fs, and p.T1478fs), 4 nonsense (p.E1198X, p.K1292X, p.E1361X, and p.E1379X), and 1 missense (p.L1493I) mutations. Interestingly, one patient with low grade adenoma and one patient with carcinoma had two concurrent *APC* deleterious mutations (2 insertions (p.G1481fs and p.E1536fs) in the adenoma sample and 1 deletion (p.E1542fs) and 1 nonsense (p.E1379X) type of *APC* mutation in the carcinoma sample).

Interestingly, several identical *APC* mutations have been observed both in high-grade adenomas and in situ carcinomas, namely: deletion (p.T1469fs), insertion (p.Q1226fs) and nonsense (p.E1379X) mutations.

### KRAS

For the *KRAS* gene, several missense mutations were successfully identified in 34 individuals [24 individuals with either low- or high-grade adenoma and 10 with in situ carcinoma, (Fig. 1C)] at codon 12: c.34G > C/A/T, c.35G > A/T/C, codon 13: c.38G > A/C/T, codon 61: c. 182A > G, c.183A > C: and 146: c.437C > T. Both adenomas and carcinomas were most frequently mutated at codon 12, position 35 (22 cases out of 34: in particular, in 16 adenomas and 6 carcinomas, 73% and 27%, respectively) and a G > A transition was the most common nucleotide for both phenotypes (14 cases out of 34: in particular, 10 in adenomas and 4 in carcinomas, 71% and 29%, respectively). The presence of *KRAS* mutation was significantly associated with the presence of villous histology (*p* = 0.02).

### TP53

Concerning the *TP53* gene, we have focused on the entire ORF. Nine patients with adenomas and 4 carcinoma patients bore mutations in the *TP53* gene (Fig. 1D). In the adenoma samples (either grade), 5 of them had missense (p.R43H, p.P45S, p.H47R, p.R49C, and p.R116Q) mutations and 4 had insertion (twice p.H46fs and twice p.C143fs). On the other hand, 4 mutations were identified in carcinoma samples: 2 missense (p.R43H and p.I122V) and 2 insertion (p.P59fs and p.V71fs). None of the analyzed patients had more than one *TP53* mutation. The p.R43H missense mutation was observed both in high-grade adenoma and in in situ carcinoma tissues.

### *APC, KRAS *and *TP53* co-mutations

Only 4 out of 96 samples had all 3 *APC, KRAS* and *TP53* genes mutated. Interestingly, 1 sample was a carcinoma, 2 samples were high grade dysplasia adenomas, and 1 sample a low grade dysplasia adenoma (Fig. 1F,G). All above samples had an MSS status and were located in the right colon. The individual with low grade dysplasia later developed another adenoma in the bowel.

Simultaneously, when we compared the frequency of mutations in these three genes between both low- and high-grade adenomas and in situ carcinoma, the *APC* mutation frequency was lower in adenoma samples than in carcinoma samples (47.3% vs. 54.5%, *p* = 0.63). The *KRAS* mutation frequency was again lower in adenoma than in carcinoma samples (32.4% vs. 45.5%, 0.31), while the *TP53* mutations frequency was the lower in adenomas and the highest in carcinoma samples (12.2% vs. 18.2%, *p* = 0.27). These distribution differences among adenomas and in situ carcinomas were not significant. Further, when we stratified mutation frequency for low- and high-grade adenoma, the distribution was as follows: low-grade adenoma—high-grade adenomas- in situ carcinoma: *APC* gene 42.9–56.0–54.5%; *KRAS* gene 32.7–32.0–45.5%; *TP53* gene 8.2–20.0–18.2%.

### Other mutations

Four *NRAS* mutations were found, two missense (twice c.181A (p.Q61K) in the right colon and a c.38A (p.G13R)) mutations and insertion (c.430dupA:p.T144fs) in the left colon. The first two mentioned missense mutations have recently been described in adenomas^7^. The second mentioned *NRAS* mutation was observed in in situ carcinoma in our set of patients.

In the present study, *BRAF* mutation c.1799 T > A (p.V600E) was only observed in a single carcinoma sample that also displayed MSI-H status. Further, three *PIK3CA* mutations were detected, namely a hotspot missense mutation (c.1624G > A, p.E542K) in an in situ carcinoma and two deletions ((twice c.3114delT:p.Y1038fs), one in a low-grade and one in a high-grade dysplasia sample). *SMAD4* (c.941dupT:p.I314fs in high-grade dysplasia and c.353_354insA:p.A118fs in low-grade dysplasia) and *PTEN* (twice c.908dupT:p.I303fs in low- and high-grade dysplasia) mutations were also recorded.

Additionally, mutations in *POLD1* and *POLE* genes were studied. For the *POLD1* gene, a c.1263 dupG:p.L421fs mutation was observed in low grade dysplasia. Interestingly, in 12 samples (11 adenomas and 1 in situ carcinoma, (Fig. 1E), *POLE* (c.T1166C:p.F389S) mutation was identified. However, no record about this particular mutation was published in COSMIC^10^, LOVD^11^ or HGMD^12^. For this reason, we rather assessed this genetic variant as variant of uncertain significance (VUS) rather than pathogenic mutation.

There was no significant overlap between these mutations identified in our set of samples.

### Follow up

For 70 out of 96 patients included into the study, follow up data were available. Out of these, 21 developed a subsequent adenoma. Seventeen of them had previously adenoma (7 high-grade and 10 low-grade adenoma) and four in situ carcinoma (Table 2). The only common feature of these patients is that their first samples included in our study were all of an MSS status (apart one low grade adenomas that was MSI-L). Unfortunately, we were not able to obtain the following adenoma tissue for analysis. Apart from one low-grade and one high-grade adenoma, all of them had previously at least one mutation in the high-risk genes, such as *APC*, *KRAS*, or *TP53*, however no association was observed in relation of the presence of any singular mutation and occurrence of subsequent adenoma. Concerning the histology and subsequent adenoma, patients with mixed histology (tubule-villous) were less prone to develop further adenomas than those with villous or tubular histology alone (*p* = 0.02). Besides, patients with higher age also develop subsequent adenomas rather than younger patients (*p* = 0.04).

**Table 2:** Follow up of patients included into the study.

	Normal findings	Recurrence of adenoma	Occurrence of CRC
Low grade adenomas	39	10	2
High grade adenomas	18	7	1
In situ carcinomas	12	4	6

In all patients with in situ carcinoma, the tumor was surgically removed, and patients were regularly monitored by colonoscopy procedure.

Furthermore, out of 22 patients with in situ carcinoma, 4 developed adenoma (as stated above) and 6 even invasive carcinoma within few years after the first in situ carcinoma. The proportion of patients developing subsequent invasive carcinoma depended significantly on the type of their primary lesion (*p* = 0.009), being by far the highest for in situ carcinomas (27.3%) in comparison to low-grade and high-grade adenomas (4.1% and 4.0%, respectively).

Most of the patients with in situ carcinoma who underwent a clinical follow up had further developed CRC in about the same location as the previous findings quite soon or within two years.

For the patient with adenoma and subsequently developed CRC, the situation is a bit different. In a few years, the emerging CRC was located in a completely different colon segment than the previous adenoma. One patient with low grade adenoma developed liver metastases very quickly.

## Discussion

The progression from adenoma into cancer can take as long as 20 years and is not usually affected by a single pathway^5,13,14^. This transition represents a multistep process that is characterized by chromosomal instability (CIN), MSI, and CIMP. Effects of all these pathways may combine and are responsible for genetic instability in an adenoma that underlies malignant transformation^15^.

Despite this, little is known about the mutation profiles of advanced adenomas and in situ carcinomas. It is not certain whether they share the same genetic background, or the driver mutations are more abundant in in situ carcinomas compared to adenomas. As these two forms of neoplasia are described as subsequent stages of carcinogenesis in the Vogelstein model, the cascade of low grade—high grade- adenoma and in situ carcinoma represents a suitable model for the analysis of CRC development. Up to now, the research of the transformation of colon adenoma into cancer has mostly focused on association studies assessing the cancer risk or advanced colorectal carcinomas.

The aim of the present study was to use a well-defined series of adenomas and in situ carcinomas to perform a parallel investigation of the mutation status of 11 genes known to be involved in CRC, as hypothesized by Vogelstein. The studied genes are involved in several different signal transduction pathways.

According to the recent study by Lee-Six et al.^16^, mutations in *APC, KRAS* and *TP53* genes are common in CRC (accounting for 56% of base-substitution and indel driver mutations) while being rare among unaffected colonic crypts. The authors suggested that mutations in these genes confer higher likelihoods of conversion of normal epithelium to adenoma and carcinoma. However, in our study, only 4 out of 96 individuals had all *APC, KRAS* and *TP53* genes mutated concurrently suggesting that further alterations in mutational frequency and spectrum may occur along with CRC progression.

In our study, we did not observe any significant differences in the distribution frequency of mutations in *APC, KRAS* and *TP53* genes between adenoma and in situ carcinomas. Without the stratification for low- and high-grade adenoma, the mutation frequencies were rather similar. The *APC* mutation frequency was moderately lower in adenoma samples (47.3%) than in carcinoma samples (54.5%). The *KRAS* mutation frequency was again lower in adenoma than in carcinoma samples (32.4% and 45.5%), as were the *TP53* mutation frequency (12.2% and 20%). Only 4 out of 96 individuals had all *APC, KRAS* and *TP53* genes mutated concurrently. Further, when we stratified mutation frequencies for low- and high-grade adenoma, the distribution frequencies of mutations in the *APC* and *TP53* genes had an increasing tendency towards in situ carcinoma, while the frequency of mutations in the *KRAS* gene in the low- and high-grade adenoma was lower than that in in situ carcinoma. This may point to the fact that in situ carcinoma still carries the mutation profile of the adenoma and only during further progression the mutation frequencies change, or, alternatively vice versa that high-grade adenomas are already approaching in situ carcinomas with their mutation profile. Therefore, a more thorough examination and assessment of the risk of CRC in people with adenomas is needed. Nonetheless, the observed higher mutation frequency in in situ carcinomas resembles the generally accepted Fearon and Vogelstein model of carcinogenesis^4^. However, additional studies are warranted to track the dynamics of mutational in relation to the disease heterogeneity.

The rather higher *KRAS* mutation frequency than expected might be explained by the fact that the current study investigated *KRAS* mutations in codons 12, 13, 61 and 146 covering most of all reported mutations for *KRAS* in CRC, while earlier studies investigated mostly codon 12 and/or 13 of the *KRAS* gene only^17,18^.

In our study we have observed the presence of *KRAS* mutation associated with the presence of villous histology. Similar outcomes were obtained by Zauber et al.^19^. The authors hypothesized that non-mucinous and MSS CRC with wild-type *KRAS* gene may have had a mutation in the *KRAS* gene during their earlier stages, however the mutation was lost during further growth.

Over the last years, many studies have shown that germline mutations in the proofreading domains of *POLD1* and *POLE* predispose to CRC and other malignancies^20,21^. These mutations arise early in oncogenesis and serve as gatekeeper mutations—conferring a growth advantage to cellular subpopulations and driving tumor growth. Interestingly, in 12 samples (11 adenomas and 1 in situ carcinoma), *POLE* (c.T1166C:p.F389S) VUS was identified. However, no record about this identified VUS was published in public databases yet, this VUS was observed in oral squamous cell carcinoma^22^ and small cell lung cancer^23^ and thus it deserves further attention. Besides this, *APC* promoter methylation was significantly associated with the presence of *POLE* VUS. Although inactivating framshift not likely leads to a hypermutation profile, Poulos et al.^24^ observed the presence of coding mutation hotspots in *POLE*-mutant cancers at highly-methylated CpGs in the tumor-suppressor genes *APC* and *TP53.* This finding points to the links between methylation and mutations and DNA repair, and these mechanisms define a key part of the mutational background of cancer genomes.

While *BRAF* mutations are more common in MSI colorectal cancers, they are less prevalent in adenomas^25^. In our study, we have observed *BRAF* mutation only in one in situ carcinoma sample that simultaneously possessed the MSI-H phenotype. *BRAF* and *KRAS* mutations are mutually exclusive^26^, as was observed in our set of samples.

In the current study, three mutations were found in the *PI3KCA* and two mutations in the negative regulator of the PI3K-AKT pathway, the *PTEN* gene. According to the literature, the *PIK3CA* mutation frequency in CRC ranges between 10–30%^27^. However, *PIK3CA* mutations are less common in colorectal adenomas, around 3%, indicating that mutations in *PIK3CA* would generally arise later during the adenoma-carcinoma transition. Although it may seem that our results are not in complete agreement with these observations, we have detected a hotspot mutation (c.1624G > A, p.E542K) in an in situ carcinoma and two deletion (c.3114delT:p.Y1038fs), one in a low-grade, and one in a high-grade dysplasia sample. However, we focused only on in situ carcinomas and not on invasive CRC and the mutation frequency rate in adenomas is in concordance with other studies, being around 3%.

A further aim of the study was to regularly follow up all patients included into study. Unfortunately, we were not able to follow many of the patients for various reasons: patients did not attend the regular check-ups, moved away, died for other than gastrointestinal reasons, etc. Follow-up data were recorded by clinicians to determine if patients had, a) clear colonoscopy findings, b) recurrence of adenoma, and c) occurrence of CRC. Clinical follow-up of 70 patients out of 96 was performed within this study. Several patients developed a subsequent adenoma or even CRC. The only common feature of these patients is that their first samples included in our study were all of an MSS status and all of them previously had at least one mutation in the high-risk genes, such as *APC*, *KRAS*, or *TP53*.

Concerning the histology and subsequent adenoma, patients with mixed histology (tubule-villous) were less prone to develop further adenomas than those with tubular or villous histology (*p* = 0.02). The fact that patients with higher age developed subsequent adenomas rather than younger patients might be a result of more regular colonoscopy controls advised among older people.

Besides, out of 22 patients with in situ carcinoma, 4 further developed adenoma and 6 even invasive carcinoma within few years after the first in situ carcinoma diagnosis. The occurrence of in situ carcinoma is in itself a clear evidence of a malignant reversal in the body and therefore these conclusions are not so unexpected.

As it was stated earlier, mutations identified in both adenomas and in situ carcinomas are likely to represent early driver events. However, mutations present predominantly in carcinomas may indicate later driver mutations involved in tumor progression. Genes mutated in adenomas and not mutated in cancer tissue can illustrate either random mutation events that are not as important for cancer onset or rare events that have not been identified in cancers. So how do we know that the adenoma is no longer embarking on transformation into cancer and, conversely, that the cancer does not carry even the historical "adenoma" mutations? Or can it be stated that once the adenoma tissue has accumulated all the necessary mutations, it will switch to carcinoma so quickly that we do not have much chance of catching it in this transitional phase?

Of course, it is still more likely to find those driver mutations in carcinomas and rare events in adenomas, but depending on how fast such a transformation takes place, the classification of genes as potential early and late driving events can help dissect pathways involved in both tumor initiation and progression.

The weaknesses of the present study include the still limited number of patients and incomplete clinical follow-up, partly responsible for the impossibility of comparing the mutational profile of initial lesion and following lesion. Also, the used method detected the presence of the mutation in the one region of the lesion, thus the results do not reflect possible heterogeneity of the non-invasive colorectal lesions. Furthermore, only adenoma tissue was analyzed in this study. Unfortunately, due to Ethical reason, the adjacent unaffected tissue was not collected and analyzed. For this reason, we cannot say with certainty whether we have identified exclusively somatic variants.

The early cancer biomarkers are strongly needed and are taking advantage of rapid progress in molecular biology. These biomarkers should be able to distinguish healthy people from patients with adenomas and subjects with early-stage CRC (stage Tis, I or II) with relative ease and low cost, and to be minimally invasive with aim to increase screening acceptability. With this respect, circulating nucleic acid-based biomarkers (or so-called “liquid biopsy”) are currently extensively studied in cancer research. Circulating cell-free DNA (cfDNA) is probably the most promising tool among all components of liquid biopsy.

Pathogenic mutations in the *KRAS, BRAF, APC*, and *TP53* genes have been predominantly analyzed in the cfDNA isolated from CRC patients and less in patients with adenoma. The concordance of the mutations found in these genes in tumor tissue and plasmatic cfDNA was 100% (reviewed in^28^). Recently, the study by Cervena et al*.*^29^ proved the clinical relevance of *APC* and *TP53* genes especially in the light of longitudinal monitoring of CRC patients.

However, the sensitivity of cfDNA based markers for early-stage disease is lower than for advanced stages^30,31^. To our knowledge, this is problem that has not been overcome yet. Although in our intended studies we would like to address this problem and attempt to detect identified pathogenic mutations in cfDNA isolated from both plasma and stool of patients with adenoma and early cancer stages.

## Conclusion

Our data confirms the Vogelstein's theory of gradual accumulation of mutations, especially in the driver genes, such as *APC*, *TP53* and *KRAS.* The set of adenomas and in situ carcinomas only very rarely exhibited MSI-H phenotype and the role of mutations in *POLE* and *PI3KCA* genes in adenoma to carcinoma transition warrants further investigations.

## Material and methods

### Tissue samples

Fresh frozen tissue samples from adenomas and in situ carcinomas were collected consecutively at three different institutes during the planned colonoscopy (Thomayer University Hospital, University Hospital Kralovske Vinohrady, and Mediconas, all in Prague, Czech Republic). The study included individuals with adenomas of tubular, villous or tubulo-villous histology and individuals with in situ carcinomas who underwent colonoscopy examination as a part of CRC screening or for intestinal symptoms. There were no age, gender, and ethnicity restrictions. The exclusion criteria were proven hereditary CRC syndromes, inflammatory bowel disease (IBD), histology of hyperplastic polyps, and size smaller than 5 mm. Patients with any personal history of previous malignancy, or with colorectal cancer-associated well-defined inherited syndromes (including Lynch syndrome, familial adenomatous, and MUTYH-associated polyposis) were also excluded from the study.

The study was approved by Ethical committees of all institutions (Institute of Experimental Medicine, Prague, Czech Republic, IKEM and Thomayer hospital) and all individuals agreed with participation in the study and signed an informed consent in accordance with the World Medical Association Declaration of Helsinki.

Colorectal adenomas were histologically classified according to the revised Vienna classification^32^. Low-grade dysplasia was marked Category 3, while category 4.1 was assigned to high-grade dysplasia. Accordingly, category 4.2 was as assigned to carcinomas in situ. Adenomas with dysplasia (categories 3 and 4.1) or with carcinoma (other categories, such as 4.2) were analyzed separately.

All patients were monitored with a regular follow-up until December 31, 2019. Follow-up data were recorded by clinicians to determine if patients had normal findings on follow-up colonoscopy, recurrence of adenoma, or occurrence of CRC.

### DNA and RNA isolation and quality control

Total DNA was isolated using AllPrep DNA/RNA Isolation kit according to the manufacturer’s protocol (Qiagen, Germany). Quantity and purity of DNA was measured using Nanodrop. OD260/280 ratios of all samples ranged between 1.8 and 2.0. After the isolation, DNA was stored at -80 °C.

### Bisulfite modification

200 ng of DNA from each sample were treated with sodium bisulfite using the ‘‘EpiTect Bisulfite Kit’’ (Qiagen, Germany) according to the manufacturer’s protocol.

### MS-HRM

For the MS-HRM of the *APC* gene, methylation independent primers, based on Migheli et al.^33^, were employed. Primer sequences for *MLH1* gene were described earlier^34^. All analyses were run according to the following conditions: 1 cycle of 95 °C for 12 min, 60 cycles of 95 °C for 30 s, Ta for 30 s and 72 °C for 15 s; followed by an HRM step of 95 °C for 10 s and 50 °C for 1 min, 65 °C for 15 s, and continuous acquisition to 95 °C at one acquisition per 0.2 °C. PCR was performed in a final volume of 25 µl, containing 12.5 µl of master mix (Qiagen), 10 pmol of each primer and 1 µl (almost 10 ng) of bisulfite-modified DNA template. Each reaction was performed in triplicate. We analyzed 10% of the samples independently on separate occasions to verify the inter-assay variability and observed a good reproducibility.

Fully methylated and unmethylated DNA (EpiTectH methylated and unmethylated human control DNA, bisulfite converted, Qiagen, Germany) were mixed to obtain the following ratios of methylation: 0%, 12.5%, 25%, 50%, 75%, 100%. Standard curves with known methylation ratios were included in each assay and were used to deduce the methylation ratio of each tumor and reference sample.

### MSI Status

MSI status was determined by molecular testing of five mononucleotide repeat markers (Bethesda consensus panel, BAT-25, BAT-26, NR-21, NR-24, and NR-27) that were run as a pentaplex, using fluorescently labeled primers and standard PCR as described in Kroupa et al.^35^. Fragment analysis was performed on ABI 3130 (Applied Biosystems). A comparison between the adenoma or in situ carcinoma and adjacent mucosa DNA short tandem repetition profiles were analyzed with GeneMapper v4.1 software (Applied Biosystems). When one or more markers were instable, the sample was interpreted as MSI, all other samples were classified as microsatellite stable (MSS). Further, when one instable marker was presented, the sample was indicated as MSI-Low (MSI-L), in the case that 2 or more markers were instable, the sample was marked as MSI-High (MSI-H).

### Mutation analysis

DNA concentrations were measured prior to amplification, using the Qubit® dsDNA HS assay (Life Technologies) and diluted to a concentration of 5 ng/μl.

Mutations were determined using a custom multiplex PCR sequencing panel consisted of M13-tailed primer pairs, as described previously^36^. The custom primers cover mutational hotspots in the genes *BRAF, EGFR, KRAS, NRAS, PIK3CA* and SMAD4, the exonuclease domain of *POLE* and *POLD1* and the entire coding sequence of *APC, PTEN and TP53* (primer sequences available upon request).

PCR was performed with FastStart Hifi Enzyme Blend (Sigma-Aldrich, St. Louis, MO, USA) in two PCR pools with non-overlapping M13-tailed primers. PCR products per sample were combined and purified with Agencourt AMPure XP beads (Beckman Coulter Life Sciences, Brea, CA, USA). In a second PCR barcodes and sequencing primers A and P1 were added. The reads generated by the Ion Torrent PGM sequencer (Thermo Fisher) were mapped against the human reference genome (GRCh37/hg19) using the TMAP 5.0.7 software with default parameters (https://github.com/iontorrent/TS). Variants were called with VarScan with more conservative coverage and minimum variant allele frequency cut-off values for indels (min-coverage = 20, min-var-freq = 0.2) than for single nucleotide variants (min-coverage = 8, min-var-freq = 0.1).

### Statistical analysis

The occurrence of individual mutations (independently) between groups was assessed by the Fisher's exact test. Similarly, their possible associations with the localization, gender, or histology type and mutually with each other were determined by the Fisher's exact test. Associations of mutations with age, Vienna classification and APC methylation were analyzed using the Mann–Whitney *U* test. The statistical analysis was performed using STATISTICA (version 11Cz; TIBCO Software Inc., Palo Alto, CA, USA), Matlab (version 2019b; The MathWorks, Inc., Natick, MA, USA), SISA (https://www.quantitativeskills.com/sisa/statistics/fiveby2.htm) and JVenn (http://jvenn.toulouse.inra.fr/app/index.html,^37^). Confidence intervals of mutation frequencies were calculated according to Agresti and Coull^38^. All reported p-values are two-tailed and the level of statistical significance was set at α = 0.05.

## Supplementary Information

Supplementary Table.
