# Author Correction: Mutational analysis of driver genes defines the colorectal adenoma: in situ carcinoma transition

**DOI:** 10.1038/s41598-022-09561-7

**Published:** 2022-04-04

**Authors:** Jiri Jungwirth, Marketa Urbanova, Arnoud Boot, Petr Hosek, Petra Bendova, Anna Siskova, Jiri Svec, Milan Kment, Daniela Tumova, Sandra Summerova, Zdenek Benes, Tomas Buchler, Pavel Kohout, Tomas Hucl, Radoslav Matej, Ludmila Vodickova, Tom van Wezel, Pavel Vodicka, Veronika Vymetalkova

**Affiliations:** 1 Institute of Biology and Medical Genetics, Institute of Physiology, 1st Faculty of Medicine, Charles University, Albertov 4, 128 00 Prague, Czech Republic; 2 Department of Surgery, Weiden Clinic, Söllnerstraße 16, 92637 Weiden in Der Oberpfalz, Germany; 3 Department of Molecular Biology of Cancer, Institute of Experimental Medicine of the Czech Academy of Sciences, Videnska 1083, 142 00 Prague, Czech Republic; 4 Department of Pathology, Leiden University Medical Center, Leiden, The Netherlands; 5 Biomedical Center, Faculty of Medicine in Pilsen, Charles University, Alej Svobody 76, 323 00 Pilsen, Czech Republic; 6 Institute of Molecular Genetics of the Czech Academy of Sciences, Videnska 1083, 142 20 Prague, Czech Republic; 7 Department of Radiotherapy and Oncology, Third Faculty of Medicine, Charles University, Srobarova 50, 100 34 Prague 10, Czech Republic; 8 Second Department of Internal Medicine, Third Faculty of Medicine, Charles University, Srobarova 50, 100 34 Prague 10, Czech Republic; 9 DT Gastroenterology, Roskotova 1/1225, Prague 4, Czech Republic; 10 Department of Internal Medicine, Third Faculty of Medicine Charles University and Thomayer University Hospital, Ruska 87, 100 00 Prague, Czech Republic; 11 Department of Oncology, First Faculty of Medicine, Charles University and Thomayer University Hospital, Videnska 800, 140 59 Prague, Czech Republic; 12 Department of Hepatogastroenterology, Institute for Clinical and Experimental Medicine, Videnska 1958/9, 140 21 Prague, Czech Republic; 13 Department of Pathology and Molecular Medicine, Third Faculty of Medicine, Charles University and Thomayer University Hospital, Videnska 800, 140 59 Prague, Czech Republic; 14 Department of Pathology, Third Faculty of Medicine, Charles University and University Hospital Kralovske Vinohrady, Srobarova 50, 100 34 Prague 10, Czech Republic

Correction to: *Scientific Reports* 10.1038/s41598-022-06498-9, published online 16 February 2022

The original version of this Article contained errors in the spelling of the authors Jiri Jungwirth, Marketa Urbanova, Arnoud Boot, Petr Hosek, Petra Bendova, Anna Siskova, Jiri Svec, Milan Kment, Daniela Tumova, Sandra Summerova, Zdenek Benes, Tomas Buchler, Pavel Kohout, Tomas Hucl, Radoslav Matej, Ludmila Vodickova, Tom van Wezel, Pavel Vodicka & Veronika Vymetalkova which were incorrectly given as Jungwirth Jiri, Urbanova Marketa, Boot Arnoud, Hosek Petr, Bendova Petra, Siskova Anna, Svec Jiri, Kment Milan, Tumova Daniela, Summerova Sandra, Benes Zdenek, Buchler Tomas, Kohout Pavel, Hucl Tomas, Matej Radoslav, Vodickova Ludmila, van Wezel Tom, Vodicka Pavel & Vymetalkova Veronika respectively.

The original Article has been corrected.

