# Supplementary material for: Mutational analysis of driver genes defines the colorectal adenoma: in situ carcinoma transition
Source: Sci Rep. 2022 Feb 16;12:2570. doi: 10.1038/s41598-022-06498-9 (PMC8850440; doi:10.1038/s41598-022-06498-9)
Supplement: Supplementary file 1 — Supplementary Table. [file 41598_2022_6498_MOESM1_ESM.docx]

**Correction: Mutational analysis of driver genes defines the colorectal adenoma: in situ carcinoma transition**

Jungwirth Jiri^1,2+^, Urbanova Marketa^1,3+^, Boot Arnoud^4^, Hosek Petr^5^, Bendova Petra^3,5^, Siskova Anna^1,3^, Svec Jiri^6,7^, Kment Milan^8^, Tumova Daniela^9^, Summerova Sandra^10^, Benes Zdenek^10^, Buchler Tomas^11^, Kohout Pavel^10^, Hucl Tomas^12^, Matej Radoslav^13,14^, Vodickova Ludmila^1,3,5^, van Wezel Tom^4^, Vodicka Pavel^1,3,5^, Vymetalkova Veronika^1,3,5^*****

1 Institute of Biology and Medical Genetics, Institute of Physiology, 1^st^ Faculty of Medicine Charles University, Albertov 4, 128 00 Prague, Czech Republic

2 Department of surgery, Weiden Clinic, Söllnerstraße 16, 92637 Weiden in der Oberpfalz, Germany

3 Department of Molecular Biology of Cancer, Institute of Experimental Medicine of the Czech Academy of Sciences, Videnska 1083, 142 00 Prague, Czech Republic

4 Department of Pathology, Leiden University Medical Center, Leiden, The Netherlands.

5 Biomedical Center, Faculty of Medicine in Pilsen, Charles University, Alej Svobody 76, 323 00 Pilsen, Czech Republic

6 Institute of Molecular Genetics of the Czech Academy of Sciences, Videnska 1083, 142 20, Prague, 4, Czech Republic

7 Department of Radiotherapy and Oncology, Third Faculty of Medicine, Charles University, Prague, Srobarova 50, 100 34, Prague, 10, Czech Republic

8 Second Department of Internal Medicine, Third Faculty of Medicine, Charles University, Prague, Srobarova 50, 100 34, Prague, 10, Czech Republic

9 DT Gastroenterology, Roskotova 1/1225, Prague 4, Czech Republic

10 Department of Internal Medicine, Third Faculty of Medicine Charles University and Thomayer University Hospital, Ruska 87, 100 00, Prague, Czech Republic

11 Department of Oncology, First Faculty of Medicine, Charles University and Thomayer University Hospital, Videnska 800, 140 59 Prague, Czech Republic

12 Department of Hepatogastroenterology, Institute for Clinical and Experimental Medicine, Videnska 1958/9, 140 21, Prague, Czech Republic

13 Department of Pathology and Molecular Medicine, Third Faculty of Medicine, Charles University and Thomayer University Hospital, Videnska 800, 140 59 Prague, Czech Republic

14 Department of Pathology, Third Faculty of Medicine, Charles University and University Hospital Kralovske Vinohrady, Srobarova 50, 100 34, Prague, 10, Czech Republic

*****Veronika Vymetalkova

Department of Molecular Biology of Cancer

Videnska 1083, 142 20 Prague, Czech Republic.

Tel. +420 2 296 4 2699

Fax. +420 2 410 6 2782

e-mail: [veronika.vymetalkova@iem.cas.cz](mailto:veronika.vymetalkova@iem.cas.cz)

**Keywords**: colorectal adenoma, in situ carcinoma, mutation, methylation, follow-up

+ The authors contributed equally.

Following the original article [1], the authors identified a misunderstanding regarding not stating the correct NM for mutations in *APC* and *TP53* genes. In the publication sometimes noncanonical NM was used instead of canonical NM. To avoid misleading, here, we provide Table 1 with both nomenclatures to better assign observed mutations.

**Table 1.** List of observed mutations in *APC* and *TP53* genes with noncanonical and canonical NM in hg19.

| In order as published in [1] | Codon position | Amino acid change | Codon position | Amino acid change |
| --- | --- | --- | --- | --- |
| ***APC* gene** | **NM_001127511** | | **NM_000038** | |
| ***Adenoma samples*** | | | | |
| **Deletions** | | | | |
| p.E1268fs | c.3803delA | p.E1268fs | c.3857delA | p.E1286fs |
| p.1275_1275del | c.3823_3824del |  | c.3877_3878del |  |
| p.D1279fs | c.3837delT | p.D1279fs | c.3891delT | p.D1297fs |
| p.T1283fs | c.3848delC | p.T1283fs | c.3902delC | p.T1301fs |
| p.1289_1291del | c.3867_3871del |  | c.3921_3925del |  |
| p.1302_1304del | c.3906_3910del |  | c.3960_3964del |  |
| p.1344_1350del | c.4031_4050del |  | c.4085_4104del |  |
| p.P1406fs | c.4216delC | p.P1406fs | c.4270delC | p.P1424fs |
| p.1431_1432del | c.4292_4295del |  | c.4346_4349del |  |
| p.T1469fs | c.4406delC | p.T1469fs | c.4460delC | p.T1487fs |
| p.P1479fs | c.4435delC | p.P1479fs | c.4489delC | p.P1497fs |
| p.1561_1562del | c.4682_4685del |  | :c.4736_4739del |  |
| **Insertions** | | | | |
| p.Q1226fs | c.3677dupA | p.Q1226fs | c.3731dupA | p.Q1244fs |
| p.S1316fs | c.3947dupC | p.S1316fs | c.4001dupC | p.S1334fs |
| p.V1359fs | c.4076dupT | p.V1359fs | c.4130dupT | p.V1377fs |
| p.P1391fs | c.4171_4172insA | p.P1391fs | c.4225_4226insA | p.P1409fs |
| p.T1469fs |  |  |  |  |
| p.G1481fs | c.4444dupT | p.G1481fs | c.4498dupT | p.G1499fs |
| p.E1536fs | c.4607dupA | p.E1536fs | c.4661dupA | p.E1554fs |
| **Nonsense mutations** | | | | |
| p.Q1273X | c.C3817T | p.Q1273X | c.C3871T | p.Q1291X |
| p.Q1349X | c.C4045T | p.Q1349X | c.C4099T | p.Q1367X |
| p.E1379X | c.G4135T | p.E1379X | c.G4189T | p.E1397X |
| p.R1432X | c.C4294T | p.R1432X | c.C4348T | p.R1450X |
| p.Q1276X | c.C3826T | p.Q1276X | c.C3880T | p.Q1294X |
| p.E1288X | c.G3862T | p.E1288X | c.G3916T | p.E1306X |
| p.S1297X | c.C3890A | p.S1297X | c.C3944A | p.S1315X |
| p.E1304X | c.G3910T | p.E1304X | c.G3964T | p.E1322X |
| p.E1335X | c.G4003T | p.E1335X | c.G4057T | p.E1353X |
| p.Q1349X | c.C4045T | p.Q1349X | c.C4099T | p.Q1367X |
| **Missense mutations** | | | | |
| p.T1274M | c.C3821T | p.T1274M | c.C3875T | p.T1292M |
| p.E1299Q | c.G3895C | p.E1299Q | c.G3949C | p.E1317Q |
| ***In situ carcinoma samples*** | | | | |
| **Deletions** | | | | |
| p.1284_1286del | c.3852_3858del |  | c.3906_3912del |  |
| p.1454_1455del | c.4361_4364del |  | c.4415_4418del |  |
| p.T1469fs | c.4407delT | p.T1469fs | c.4461delT | p.T1487fs |
| p.E1542fs | c.4625delA | p.E1542fs | c.4679delA | p.E1560fs |
| **Insertions** | | | | |
| p.Q1226f | c.3677dupA | p.Q1226fs | c.3731dupA | p.Q1244fs |
| p.M1365fs | c.4096dupT | p.M1365fs | c.4150dupT | p.M1383fs |
| p.S1377fs | c.4129_4130insTATC | p.S1377fs | 4183_4184insTATC | p.S1395fs |
| p.T1478fs | c.4435dupC | p.T1478fs | c.4489dupC | p.T1496fs |
| **Nonsense mutations** | | | | |
| p.E1198X | c.G3592T | p.E1198X | c.G3646T | p.E1216X |
| p.K1292X | c.A3874T | p.K1292X | c.A3928T | p.K1310X |
| p.E1361X | c.G4081T | p.E1361X | c.G4135T | p.E1379X |
| p.E1379X | c.G4135T | p.E1379X | c.G4189T | p.E1397X |
| **Missense mutations** | | | | |
| p.L1493I | c.C4477A | p.L1493I | c.C4531A | p.L1511I |
|  |  |  |  |  |
| ***TP53* gene** | **NM_001126115** | | **NM_000546** | |
| ***Adenoma samples*** | | | | |
| **Missense mutations** | | | | |
| p.R43H | c.G128A | p.R43H | c.G524A | p.R175H |
| p.P45S | c.C133T | p.P45S | c.C529T | p.P177S |
| p.H47R | c.A140G | p.H47R | c.A536G | p.H179R |
| p.R49C | c.C145T | p.R49C | c.C541T | p.R181C |
| p.R116Q | c.G347A | p.R116Q | c.G743A | p.R248Q |
| **Insertions** | | | | |
| p.H46fs | c.136dupC | p.H46fs | c.532dupC | p.H178fs |
| p.C143fs | c.427dupT | p.C143fs | c.823dupT | p.C275fs |
| ***In situ carcinoma samples*** | | | | |
| **Missense mutations** | | | | |
| p.R43H | c.G128A | p.R43H | c.G524A | p.R175H |
| p.I122V | c.A364G | p.I122V | c.A760G | p.I254V |
| **Insertions** | | | | |
| p.P59fs | c.176dupC | p.P59fs | c.293dupC | p.P98fs |
| p.V71fs | c.212dupT | p.V71fs | c.608dupT | p.V203fs |
|  |  |  |  |  |

**Reference**:

[1] Jungwirth J, Urbanova M, Boot A, Hosek P, Bendova P, Siskova A, Svec J, Kment M, Tumova D, Summerova S, Benes Z, Buchler T, Kohout P, Hucl T, Matej R, Vodickova L, van Wezel T, Vodicka P, Vymetalkova V. Mutational analysis of driver genes defines the colorectal adenoma: in situ carcinoma transition. Sci Rep. 2022 Apr 4;12(1):5595. doi: 10.1038/s41598-022-09561-7. PMID: 35379882
